# Bioaffinity ultrafiltration coupled with HPLC-ESI-MS/MS for screening potential α-glucosidase inhibitors from pomegranate peel

**DOI:** 10.3389/fnut.2022.1014862

**Published:** 2022-10-18

**Authors:** Rujie Shi, Nong Zhou, Han Zhang, Min Gong, Lin Han

**Affiliations:** ^1^College of Biology and Food Engineering, Chongqing Three Gorges University, Chongqing, China; ^2^College of Food Science and Engineering, Northwest A&F University, Yangling, China

**Keywords:** pomegranate peel, ultrafiltration-HPLC-ESI-MS/MS, ellagic acid, α-glucosidase, inhibitory mechanisms

## Abstract

Pomegranate peel (PoP) contains plenty of bioactive compounds and exhibits strong activity to prevent postprandial hyperglycaemia and improve diabetes mellitus. Presently, bioaffinity ultrafiltration coupled with high performance liquid chromatography-electrospray ionization mass spectrometry (HPLC-ESI-MS/MS) is employed to screen and identify the efficient α-glucosidase inhibitors in PoP and the detailed inhibitory mechanisms are further investigated. The results show that many substances, including ellagic acid, kaempferol, gallic acid, and resveratrol in PoP reveal strong activity to inhibit α-glucosidase and ellagic acid (EA) is screened as the most effective compound. Further research indicates that EA plays a competitive and reversible inhibition role against α-glucosidase with the value of *K*i was 6.24 × 10^5^ mol/L. EA also directly interacts with the amino acids of α-glucosidase mainly *via* van der Waals forces and hydrogen bonds, thereby, influencing the secondary structure and stability of α-glucosidase. Finally, the α-glucosidase inhibitory activity of EA is further confirmed to significantly reduce postprandial blood glucose *in vivo*.

## Introduction

Diabetes mellitus is one of the most serious chronic metabolic diseases all over the world with the typical characteristic of hyperglycemia (1). Inhibiting the digestion and absorption of carbohydrates plays a key role in controlling postprandial blood glucose and preventing hyperglycemia, which is closely related with the activity of carbohydrate digestive enzymes, such as α-amylase, α-glucosidase, etc. (2). Reducing the activity of α-glucosidase can effectively prevent the release of D-glucose from the non-reducing end side of disaccharide, thus, suppressing the generation of glucose and ultimately decreasing the postprandial hyperglycemia (3). Compared to acarbose, a commercial α-glucosidase inhibitor, many natural compounds in fruits and vegetables effectively inhibited α-glucosidase with fewer side effects (4). Therefore, screening and identification natural α-glucosidase inhibitors with stronger activity will provide us more methods and choices to prevent postprandial hyperglycemia and ameliorate diabetes mellitus.

Pomegranate (*Punica granatum* L.), an ancient and popular fruit, is widely cultivated in the subtropical and tropical regions of the world (5). As a byproduct, PoP comprises about 30–40% portion of the fruit with plenty of bioactive compounds (6). Previous reports demonstrated that PoP contained lots of high molecular weight phenolics, ellagitannins, proanthocyanidins, flavonoids, and complex polysaccharides; especially the phenolic content was much higher than that of any other anatomical part of the fruit (7–9). PoP has demonstrated many bioactivities, including antioxidant, antidiabetic, anticancer, and cardiovascular protection (10–12). Both PoP powder and extract displayed effective activity against diabetes, which might be tightly related with the inhibitory capacity of α-glucosidase (6, 13). Further research found that the methanolic or water extract of PoP could significantly inhibit α-glucosidase (14–16). Although some reports indicated that the α-glucosidase inhibitory activity of PoP was associated with some phenolic compounds, such as gallic acid and ellagic acid, the specific basis for PoP contributing to the inhibition of α-glucosidase has not been fully elucidated. Bioaffinity ultrafiltration combines with HPLC-MS/MS has been developed as one of the most powerful strategies to high-throughput screen and identify the bioactive components from extract. Briefly, the target protein is regarded as the receptor of the bioactive molecules (ligands), which could separate the combined ligands from the extract *via* the bioaffinity. After ultrafiltration, the ligands are released from the receptor, following further identification and quantification by HPLC-MS/MS assay (17). Wang et al. (18) has employed α-glucosidase as the receptor to screening for the potential inhibitors from Guava leaves tea (GLT) through bioaffinity ultrafiltration-HPLC-MS/MS method and found that quercetin and procyanidin B3 were the primarily responsible for the antihyperglycemic effect of GLT (18).

Therefore, the present study aimed to screen and identify the natural α-glucosidase inhibitors in PoP by using the method of bioaffinity ultrafiltration coupled with HPLC-ESI-MS/MS. After that, the interaction mechanism of screened inhibitors with α-glucosidase was further studied using enzyme kinetics, spectroscopic analysis (including fluorescence spectra and CD spectra), molecular docking, and atomic force microscopy (AFM). Moreover, a sucrose-loading test was employed to evaluate the α-glucosidase inhibitory activity *in vivo*. The results of this work provided much more information about the substances in pomegranate peel that contributed to the inhibitory activity against α-glucosidase, and uncovered interaction mechanism details between an inhibitor and α-glucosidase.

## Materials and methods

### Materials and chemicals

Pomegranate peel was purchased from Hebei Kang’an Biotech. Co. (Anguo, China). *p*-Nitrophenyl-α-D-glucopyranoside (*p*NPG) and α-glucosidase (100 U/mg) were obtained from Sigma-Aldrich (Shanghai, China). Acarbose, ellagic acid (EA), and the other compounds including nicotinic acid, isoguanosine, sclareol glycol, triptolide, sclareolide, *p*-hydroxybenzoic acid, kaempferol, gallic acid, quercetin, and resveratrol were purchased from Yuanye Biotech. Co. (Shanghai, China). All other chemicals used in this study were analytical grade.

### Preparation of different solvent extracts from pomegranate peel

20 g of PoP powder was mixed with 80 mL different solvents including distilled water (water extract), 80% (V/V) ethanol (ethanol extract), and 30% (V/V) acetone (acetone extract), respectively. The mixtures were ultrasonic-assisted extracted (80 kHz) under 40°C for 20 min. After that, the extracts were centrifuged for 10 min at 5000 r/min and the supernatant was collected. The residues were extracted twice according to the above procedures and all supernatants were dried in a vacuum freeze dryer after rotary evaporation treatment. The dried samples were sealed and preserved at −20°C. Different concentrations of extracts were prepared to determine the inhibitory activity against α-glucosidase (19).

### Ultrafiltration test and HPLC-ESI-MS/MS analysis

Ultrafiltration was performed according to previous reports with some modifications (18, 20). The dried acetone extract of pomegranate peel was re-dissolved in ammonium acetate buffer (pH = 6.8) to the concentration of 2 mg/ml. 200 μL of the sample was added to equal α-glucosidase (0.5 U/ml, dissolved in 0.1 M PBS with pH 6.8) and the mixture was incubated on the constant temperature shaker (500 r/min) at 37°C for 30 min. After that, the mixture was transferred to ultrafiltration tubes (10 kDa, Millipore) and centrifuged at 10000 r/min for 15 min. This process was repeated for 3 times by adding another 100 μl of ammonium acetate buffer to remove the unbound substances. And then, 100 μl of methanol-water solution (pH = 3.3, V/V = 1:1) was added and slightly shook before centrifuging at 10000 r/min for 15 min. This process was repeated for another 3 times and the filtrate was collected and dried in vacuum freeze dryer.

High resolution ion mobility liquid chromatography-mass spectrometry (HRLC-MS, AB SCIEX, Singapore) equipped with a Zorbax Eclipse Plus C_18_ column (250 mm × 4.6 mm, 5 μm) and high resolution tandem quadrupole time of flight mass spectrometry was employed to identify the substances in the collected samples (18). The conditions were as follows: mobile phase A was consisted with 0.1% formic acid-water (V/V), mobile phase B was acetonitrile. The elution was 0–5 min, 15% B; 5–10 min, 15–20% B; 10–20 min, 20–25% B; 20–30 min, 25–35% B; 30–40 min, 35–50% B; 40–45 min, 80% B; 45-50 min, 15% B. The flow rate was 0.8 mL/min with the injection volume of 20 μL. The mass spectrometry conditions: IDA acquisition mode; scanning range is 100–2000 (MS), 50–2000 (MS/MS); ESI (negative); temperature of ion source, 550°C; spray voltage, −4500 V.

### Inhibitory effect against α-glucosidase

Different concentrations (0.01, 0.1, 1, 5, and 10 mg/mL) of Pop extract samples, PBS (pH = 6.8), and α-glucosidase (5 U/mL) were mixed together with equal volumes and co-incubated at 37°C for 15 min. 20 μL *p*NPG (3 mM) was added to incubate for another 10 min and 150 μL Na_2_CO_3_ (0.1 mol/L) was used to stop the reaction. Finally, the absorbance was measured at 405 nm by microplate reader (Perkin Elmer, Waltham, MA, USA). Meanwhile, the blank group without samples were simultaneously carried out and the inhibition ratios were calculated (21, 22).

For identified compounds, two concentrations (0.1 mg/mL and 0.5 mg/mL) of each inhibitor were prepared and added to PBS (pH = 6.8) and α-glucosidase (5 U/mL) with equal volumes. After that, 20 μL *p*NPG (3 mM) was added to initiate the reactions and the changes of absorbance were recorded every 20 s immediately.

### Kinetic analysis

Lineweaver–Burk plot was employed to determine the inhibitory type of EA against α-glucosidase (23). Briefly, 20 μL α-glucosidase (5 U/mL) was mixed with different concentrations (0, 10, 15, 20, 30 μg/mL) of EA and incubated at 37°C for 15 min. *p*NPG (1.0, 2.0, 4.0, and 8.0 × 10^–3^ mol/L) was added to initiate the reaction and the changes of absorbance were recorded every 60 s immediately. The double-reciprocal plots of reaction velocity (υ) against different concentration of *p*NPG (1/υ vs. 1/[*p*NPG]) were analyzed using the Lineweaver–Burk plot to determine inhibition constant (*K*_*i*_).

To determine the inhibitory reversibility of EA against α-glucosidase, different concentrations (0, 20, 40, 60, and 100 μg/mL) of EA were reacted with the range of 7.5–1 U/mL α-glucosidase, the absorbance was recorded every 60 s immediately after the *p*NPG (3 mM) was added (24).

### Fluorescence spectra analysis

The fluorescence spectra of α-glucosidase with or without EA were measured at three different temperatures (298, 304, and 310 K) according to the previous study (21). Exactly, 2.5 mL α-glucosidase (15 U/mL) was titrated by continuous additions of EA (5 μL each time) to reach a final concentration range from 0 to 19.89 × 10^–6^ mol/L. After 5 min of equilibration under different temperatures, the steady fluorescence emission spectra were recorded from 300 to 460 nm with an excitation wavelength of 280 nm. Additionally, the fluorescence of free EA was measured as the background. According to fluorescence spectra data, the quenching constant (*K*_*sv*_), binding constant (*K*_*a*_), number of binding sites (*n*), the binding constant of accessible fluorophores, and the values of free energy change (△*G*^0^), △*H*^0^ and △*S*^0^ were calculated according the previous reports (21, 22, 25).

### Circular dichroism spectrum analysis

The analysis of CD spectrum was performed according to the previous report with some modifications (26). Briefly, both α-glucosidase and EA were dissolved in PBS (pH = 6.8). The CD spectra of 200 μL α-glucosidase (50 U/mL) were measured freely or with different volumes (25, 50, 100 μL) of EA (2 mg/mL) by CD spectrometer (Leatherhead, Surrey, UK). The spectrum scanning range was 200–250 nm, the response time and scanning speed were 1 s and 30 nm/, respectively.

### Observation by atomic force microscopy

Equal volumes of EA (40 μg/mL) and α-glucosidase (2.0 U/mL) were co-incubated at 37°C for 20 min, and then the mixture was evenly spread on the clean mica substrate and dried at room temperature for 12 h. Finally, AFM observation was performed by a Multimode Nanoscop V (Bruker, Billerica, MA, USA) with tapping model.

### Molecular docking

The software Sybyl 2.0 was used to carry out molecular docking for further investigating the interactions between EA and α-glucosidase. The crystal structure of α-glucosidase (PDB ID: 3A4A; gi number: 411229) was searched from the Protein Data Bank (PDB). The procedures for molecular docking were according to the report of Han et al. (25).

### Sucrose-loading test

Kunming mice (male, 35–40 g) were gained from Xi’an Jiaotong University (Xi’an, China). After adaptive feeding for 1 week, the mice were divided into four groups (*n* = 6): blank group, mice were oral administrated by gavage without sucrose and inhibitors; sucrose group, mice were oral administrated by gavage with sucrose only (2 g/kg); sucrose + acarbose group, mice were oral administrated by gavage with sucrose (2 g/kg) and acarbose (50 mg/kg); sucrose + ellagic acid group, mice were oral administrated by gavage with sucrose (2 g/kg) and EA (25 mg/kg). After gavage, the blood glucose levels were measured at 0, 15, 30, 45, 60, 90, and 120 min by glucose detection kit (Robio Co., Shanghai, China). The whole process conformed to the Guide for the Care and Use of Laboratory Animals, eighth edition (ISBN 10: 0-309-15396-4) (22).

### Statistical analysis

All data were analyzed using GraphPad Prism 8.0 software by One-way ANOVA and the results were expressed as mean ± standard deviation (SD). *p* < 0.05 and *p* < 0.01 indicated significant differences.

## Results and discussion

### The α-glucosidase inhibitory effect of extracts from pomegranate peel

Previous research demonstrated that extracts from PoP showed effective activity to improve diabetes and decrease blood glucose, which might be associated with the α-glucosidase inhibition (9). In the present study, we found that the extracts from PoP by different solvents (acetone, water, and ethanol) revealed stronger inhibitory activity against α-glucosidase than that of acarbose, a commercial α-glucosidase inhibitor (Supplementary Figure 1). Moreover, acetone extract showed the best α-glucosidase inhibitory activity among the three extracts with the IC_50_ was less than 0.01 mg/mL (Supplementary Figure 1). These results were consistent with several previous reports, which demonstrated that the extracts from pomegranate, including juice, peels, seeds, and flowers, with different solvents revealed effective inhibitory activity against α-glucosidase (13, 14, 27). However, the exact substance that plays the key role for α-glucosidase inhibition is still not clear. Subsequently, we chose the acetone extract of PoP for screening the α-glucosidase inhibitors by ultrafiltration-HPLC-ESI-MS/MS.

### Filtration and identification of α-glucosidase inhibitors from pomegranate peel

**FIGURE 1:**
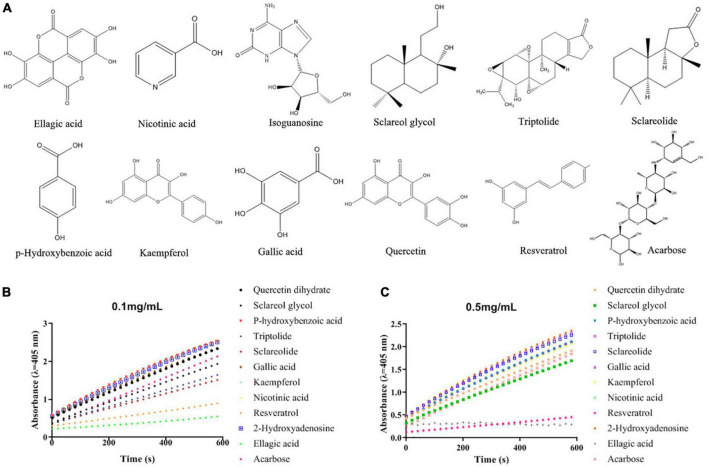
The structures of acarbose and the screened α-glucosidase inhibitors from the acetone extract of pomegranate peel **(A)** and their α-glucosidase inhibitory activities under different concentrations [**(B)** 0.1 mg/mL; **(C)** 0.5 mg/mL].

In order to screen and identify the specific α-glucosidase inhibitors, a combination of bioaffinity ultrafiltration and HPLC-ESI-MS/MS was employed in the present study. α-Glucosidase was used as an adhesion receptor for the acetone extract ligands from pomegranate peel using bioaffinity (18). After elution from the receptor, the ligands were isolated and identified by HPLC-ESI-MS/MS *via* comparing with the standards in the ESI-MS library, which was provided with the device of AB SCIEX Co. (Singapore) (Supplementary Figure 2; Table 1; 23, 28, 29). The mass spectrograms and chemical structures of the selected 11 bioactive substances were presented in Supplementary Figure 3 and their inhibitory activity against α-glucosidase were compared. As shown in Figure 1, ellagic acid (EA) exhibited the strongest activity to inhibit α-glucosidase among these identified compounds, which significantly prevented the rapid increase of absorbance at 405 nm (Figure 1). Previously, EA isolated and identified from *Rosa gallica* was reported to have almost no α-glucosidase inhibition (30). But the later articles demonstrated that EA, as hydrolysable tannins in pomegranate, revealed strong activity to inhibit α-glucosidase (14, 27). The present results further confirmed these findings. Besides EA, resveratrol also revealed strong inhibitory activity against α-glucosidase, both were stronger than acarbose (0.1 mg/mL), a commercial inhibitor, in inhibiting α-glucosidase (Figure 1). Lots of previous studies demonstrated that EA is one of the main phenols in the pomegranate, especially in the peel of a Chinese pomegranate cultivar (201.3 mg/g), but resveratrol occurs in lesser amounts if this study analyzed for stilbenes (6, 7, 31). Therefore, we next focused on EA and further investigated its inhibitory mechanisms against α-glucosidase both *in vitro* and *in vivo*.

**TABLE 1:** Identification of the main potential α-glucosidase inhibitors from the acetone extract of pomegranate peel.

No.	Compounds	Formula	Expected m/z	Found at m/z	RT* (min)	Intensity	Ref.
1	Ellagic acid	C_14_H_6_O_8_	303.0135	303.0124	7.65	2,839	Standard
2	Nicotinic acid	C_6_H_5_NO_2_	124.0393	124.0391	0.87	1,432	Standard
3	Isoguanosine	C_10_H_13_N_5_O_5_	301.2162	301.2162	0.84	1,199	Standard
4	Sclareol glycol	C_16_H_30_O_2_	255.2319	255.2319	45.27	740	Standard
5	Triptolide	C_20_H_24_O_6_	361.1646	361.165	24.62	588	Standard
6	Sclareolide	C_16_H_26_O_2_	251.2006	251.2011	45.11	494	Standard
7	*p*-Hydroxybenzoic acid	C_7_H_6_O_3_	139.039	139.0387	40.72	393	Standard
8	Kaempferol	C_15_H_10_O_6_	287.055	287.055	11.39	388	Standard
9	Gallic acid	C_26_H_34_O_5_	443.2428	443.2419	40.2	346	Standard
10	Quercetin	C_15_H_10_O_7_	303.0499	303.0502	8.69	190	Standard
11	Resveratrol	C_14_H_12_O_3_	229.0859	229.0857	41.57	163	Standard

*RT, Retention time.

### The α-glucosidase inhibitory effect of ellagic acid

**FIGURE 2:**
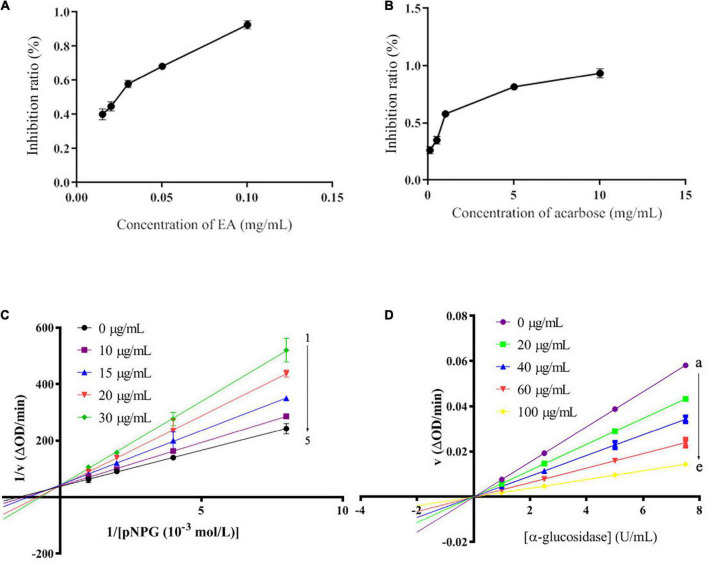
The inhibitory activity of EA **(A)** and acarbose **(B)** against α-glucosidase with different concentrations. The Lineweaver–Burk plots **(C)**, the concentrations of EA were 30, 20, 15, 10, and 0 μg/mL for curves 1→5, and the inhibitory reversibility of EA with different concentrations (0, 20, 40, 60, and 100 μg/mL for curves a→e) against α-glucosidase **(D)**.

Different concentrations of EA were prepared to further confirm its strong α-glucosidase inhibitory activity. As shown in Figure 2, EA revealed stronger capacity to inhibit α-glucosidase in a dose-dependent manner than acarbose. The IC_50_ values of EA and acarbose were 76.08 ± 0.02 μM and 1.02 ± 0.03 mM, respectively (Figures 2A,B). The number and position of hydroxyl group play an important role in the inhibition of α-glucosidase activity (4). EA have four hydroxyl groups that might closely affect its inhibitory activity. Lineweaver–Burk plots were investigated to determine the inhibitory type and the results were presented in Figure 2C. 1/[*p*NPG] showed good linearity to 1/υ with the concentrations of EA ranging from 0 to 30 μg/mL. All the straight lines intersected on Y axis, indicating that EA could competitively bind to the active site of α-glucosidase and play a competitive inhibitory role to inhibit α-glucosidase activity (Figure 2C; 32, 33). Meanwhile, the inhibition constant value (*K*i) was calculated to be 6.24 × 10^5^ mol/L. Moreover, the inhibitory reversibility of EA was researched and the result was shown in Figure 2D. EA gradually reduced the change rate of absorbance with the increase of α-glucosidase in a dose-dependent manner (Figure 2D). All the straight lines of υ vs. [α-glucosidase] passed through the original point, indicating that EA showed reversible inhibitory activity against α-glucosidase (26).

### Influence of ellagic acid on the fluorescence spectra of α-glucosidase

**FIGURE 3:**
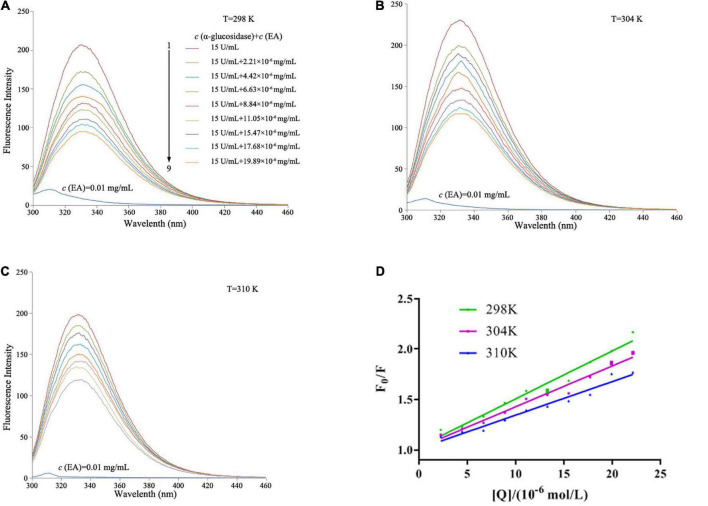
The influence of EA on the fluorescence spectra of α-glucosidase at different temperatures. **(A)** 298 K. **(B)** 304 K. **(C)** 310 K. The concentration of α-glucosidase was 15 U/mL with or without different concentrations (2.21, 4.42, 6.63, 8.84, 11.05, 15.47, 17.68, and 19.89 × 10^–6^ mol/L) of EA for the curves 1→9. EA alone almost showed no fluorescence at the concentration of 0.01 mg/mL, λ = 280 nm. **(D)** The Stern–Volmer plots of the fluorescence quenching of α-glucosidase that interacted with EA at different temperatures.

Since α-glucosidase itself has fluorescence, we investigated the influence of EA on the fluorescence intensity of α-glucosidase at three different temperatures (298, 304, and 310 K). As shown in Figure 3, free α-glucosidase revealed strong fluorescence intensity, but EA itself almost exhibited no fluorescence under different temperatures. However, the fluorescence intensity of α-glucosidase was gradually decreased with the addition of EA, indicating that EA interacted with α-glucosidase and quenched the fluorescence. Three amino acids, tyrosine, tryptophan, and phenylalanine exhibit fluorescence in the molecular structure of α-glucosidase (22). Therefore, EA might directly interact with these amino acid residues, and quench the intrinsic fluorescence of α-glucosidase.

To further exploit the quenching mechanism, *K*_*sv*_ was calculated by Stern–Volmer equation to determine the quench type, dynamic quenching or static quenching:


(1) $$\frac{F_{0}}{F}=1+K_{S\,V}\,\left[Q\right]=1+K_{q}\,\tau_{0}\,\left[Q\right]$$

where *F*_0_ and *F* are the peak of fluorescence intensities of α-glucosidase with or without EA, respectively; *K*_*sv*_ is the dynamic quenching constant; *K*q is the quenching rate constant; [Q] is the different concentrations of EA; τ_0_ is the average lifetime of fluorescence with the value of 10^–8^ s. As shown in Table 2, the values of *K*_*sv*_ were reduced with the increased temperatures, but *K*q showed greater values at each temperature than 2.0 × 10^10^ L/mol⋅s, the maximum scatter collision quenching constant, indicating that EA interacted with α-glucosidase and quenched its fluorescence by a static quenching mechanism (21).

**TABLE 2:** Quenching constants (*K*_*sv*_), binding constant (*K*_*a*_), number of binding sites (*n*), and thermodynamic parameters of the interaction between α-glucosidase and EA at different temperatures.

T (K)	*K*_*sv*_ (×10^5^ L/mol)	*K*_*a*_ (×10^3^ L/mol)	*n*	Δ*G*^0^ (kJ/mol)	Δ*H*^0^ (kJ/mol)	Δ*S*^0^ (J/mol⋅K)
298	4.75 ± 0.01	11.42 ± 0.04	0.87	−23.15 ± 0.03	−73.55 ± 0.36	−169.14 ± 0.33
304	4.05 ± 0.03	6.17 ± 0.06	0.83	−22.30 ± 0.06		
310	3.32 ± 0.01	3.76 ± 0.02	0.86	−21.12 ± 0.05		

For static quenching, the equation as follow was employed to calculate binding constant (*K*_*a*_) and number of binding sites (*n*) between EA and α-glucosidase:


(2) $$\mathrm{lg}\frac{F_{0}-F}{F}=\mathrm{lg}K_{a}+n\,\mathrm{lg}\left[Q\right]$$

With the increase of temperature, the values of *K*_*a*_ were reduced, but still exhibited strong affinity between EA and α-glucosidase (Table 2). Moreover, EA had only one binding site on the α-glucosidase as the values of *n* under different temperatures were approximately equal to 1 (Table 2).

Thermodynamic analysis was performed to further determine the binding forces between EA and α-glucosidase, and the relevant thermodynamic parameters, including△*G*^0^,△*H*^0^, and △*S*^0^, were calculated by the two following equations:


(3) $$\mathrm{lg}K_{a}=-\frac{\Delta \,H^{0}}{2.303\,R\,T}+\frac{\Delta \,S^{0}}{2.303\,R}$$

(4) $$\Delta \,G^{0}=\Delta \,H^{0}-T\,\Delta \,S^{0}$$

where *R* and *T* are the gas constant and temperature, respectively. As shown in Table 2, both the values of △*H*^0^ and △*S*^0^ were negative (−73.55 ± 0.36 kJ/mol and −169.14 ± 0.33 J/mol⋅K, respectively), indicating that van der Waals forces and hydrogen bonds were the main driving forces to promote the interaction between EA and α-glucosidase (23). In addition, all the values of △*G*^0^ were less than zero, implying that EA could spontaneously interact with α-glucosidase under the three different temperatures (21).

### Influence of ellagic acid on the circular dichroism spectra of α-glucosidase

**FIGURE 4:**
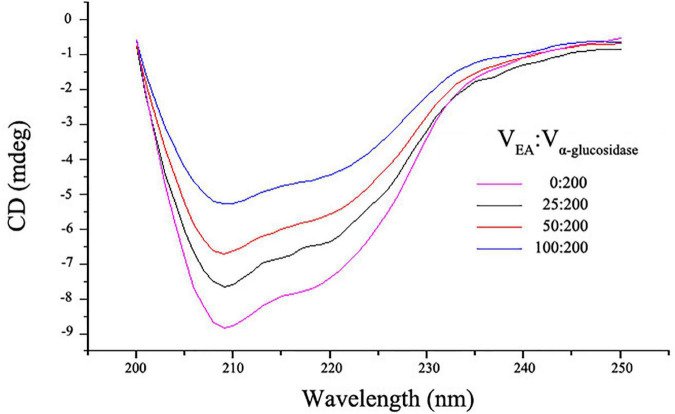
The CD spectra of α-glucosidase with or without different volumes of EA. 200 μL of α-glucosidase (5.0 U/mL) mixed with different volumes (0, 25, 50, and 100 μL) of EA (2 mg/mL) and the CD spectra was determined. The spectrum scanning range was between 200 and 250 nm.

Circular dichroism spectra were employed to further investigate the influence of EA on the secondary structures of α-glucosidase. As shown in Figure 4, free α-glucosidase has two characteristic bands of α-helix structure in the ultraviolet region at around 209 and 222 nm (23). However, the CD intensities at these two bands were decreased after adding different volumes of EA with the concentration of 2 mg/mL, indicating that the interactions between EA and α-glucosidase significantly altered the enzyme’s secondary structures (Figure 4). These observations were further supported by the quantitative calculation of the secondary structure contents (Table 3). With the addition of EA, the contents of α-helix in α-glucosidase were obviously reduced from 34.90 to 24.83%, but the β-sheet and random coil were significantly increased (from 18.37 to 31.00% and from 25.60 to 32.87%, respectively) and β-turn was slightly influenced. These results further confirmed that EA directly interacted with α-glucosidase and inhibited its activity (34).

**TABLE 3:** The secondary structure contents of α-glucosidase that influenced by different contents of EA.

V_EA_:V_α–glucosidase_	α–helix (%)	β–sheet (%)	β–turn (%)	Random coil (%)
0:200	34.90 ± 0.54	18.37 ± 0.21	17.43 ± 0.17	25.60 ± 0.22
25:200	29.53 ± 0.29	23.73 ± 0.21	18.07 ± 0.21	28.43 ± 0.21
50:200	28.17 ± 0.21	26.60 ± 0.22	18.57 ± 0.29	30.47 ± 0.33
100:200	24.83 ± 0.29	31.00 ± 0.54	18.70 ± 0.33	32.87 ± 0.33

The concentrations of EA and α-glucosidase were 2 mg/mL and 5.0 U/mL, respectively.

### Atomic force microscopy studies

**FIGURE 5:**
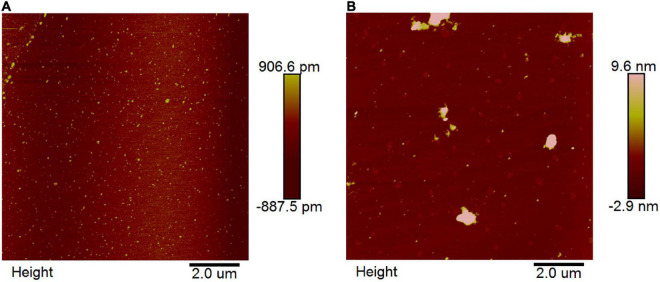
AFM images of free α-glucosidase **(A)** and the interaction between EA and α-glucosidase **(B)**. The concentrations of α-glucosidase and EA were 2.0 U/mL and 40 μg/mL, respectively. EA and α-glucosidase were co-incubated for 30 min at 37°C before preparing on the mica plate for observation.

Atomic force microscopy is a powerful imaging platform to investigate the interaction of small molecules and protein *via* visualized and manipulated ways (35). Here, we used the tapping model of AFM to study the interaction between EA and α-glucosidase. The results showed that 2.0 U/mL of free α-glucosidase was uniformly distributed on the mica plate (white spots) (Figure 5A). However, after co-incubation with 40 μg/mL of EA for 30 min, the molecules of α-glucosidase were aggregated together and formed irregular polymers (Figure 5B), indicating that EA interacted with α-glucosidase and changed its stability *via* influencing the microenvironment and conformation (21). Therefore, the unstable molecules of α-glucosidase gathered together to form much more stable polymers.

### Molecular docking analysis

**FIGURE 6:**
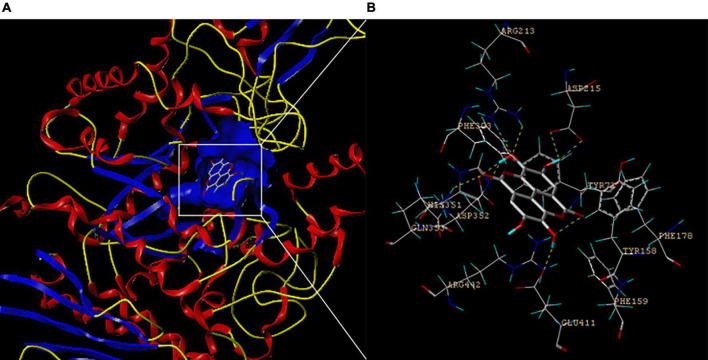
The result of molecular docking between EA and α-glucosidase. **(A)** The active cavity of α-glucosidase was occupied by EA and **(B)** these interactions mainly depended on intermolecular hydrogen bonding (dashed lines).

Molecular docking is popularly applied to investigate the precise interaction between small molecular compounds and target proteins (36). As shown in Figures 6A,B, EA inserted into α-glucosidase and occupied the bioactive site, which depended mainly on intermolecular hydrogen bond (dashed lines). These interactions occurred between EA and the amino acid residues, including Tyr72, Phe159, Phe178, Arg213, Asp215, His351, Asp352, Gln353, Glu411, and Arg442, with the average bond length of 2.23 nm and binding energy of −6.7656 kcal/mol (Figures 6A,B). The hydroxyl groups of EA played a key role in the formation of hydrogen bonds with the amino acid residues of α-glucosidase. These results indicated that EA could effectively interact with α-glucosidase at the bioactive site and inhibit catalyzing disaccharides into monosaccharide.

### Ellagic acid reduces the postprandial blood glucose

**FIGURE 7:**
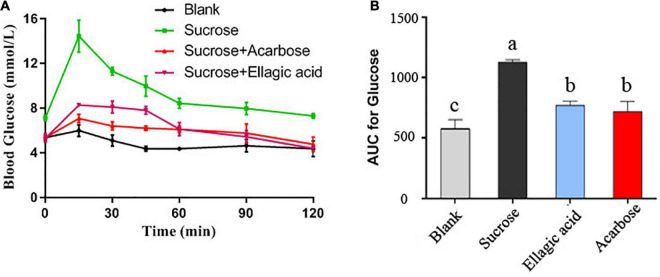
The effect of EA and acarbose on the blood glucose after intragastric administration of sucrose **(A)** and the area under curve (AUC) of blood glucose were calculated **(B)**. Different letters mean *p* < 0.05.

To further verify the inhibitory activity of EA against α-glucosidase *in vivo*, a sucrose-loading test was employed to determine the effect of EA on postprandial blood glucose. After intragastric administration with sucrose or sucrose plus EA, the blood glucose of Kunming mice was measured at 0, 15, 30, 45, 60, 90, and 120 min, respectively. As presented in Figure 7, the level of blood glucose in blank group that gavaged with 0.5% CMC-Na solution showed no significant differences from 0 to 120 min (Figures 7A,B). But, intragastric administration with sucrose only acutely increased the blood glucose of Kunming mice with the maximum appeared at 15 min. Comparatively, EA (25 mg/kg) and acarbose (50 mg/kg) obviously inhibited the rapid rise of blood glucose after the sucrose gavage, and the values of AUC were reduced 31.8 and 36.4%, respectively (Figures 7A,B). However, the concentration of EA used in this test is only half of acarbose, indicating that EA revealed better hypoglycemic activity than that of acarbose, but this result still needs further clinical verification. Sucrose is mainly catalyzed into glucose by α-glucosidase in the brush-border surface membrane of intestinal cells and the glucose is absorbed and transported into the blood (25). Hence, inhibiting the activity of α-glucosidase can effectively reduce the generation of glucose and decrease postprandial blood glucose. The current results provided more evidence to support EA as a significant inhibitor of the α-glucosidase activity *in vivo*.

## Conclusion

Much research has demonstrated that pomegranate exhibits strong antidiabetic activity and effectively decreases the blood glucose levels (11). Further investigations have found that extracts from different parts of pomegranate, including juice, peels, seeds, and flowers, reveal efficaciously α-glucosidase inhibitory activity, which reduce postprandial blood glucose. In this current study, we found that the acetone extract of pomegranate peel showed much better α-glucosidase inhibitory activity than water and ethanol extracts (Supplementary Figure 1). Eleven substances were screened and identified as the main α-glucosidase inhibitors in pomegranate peel by ultrafiltration-HPLC-ESI-MS/MS technology, and EA was found to have the strongest α-glucosidase inhibitory activity among the eluted identified compounds.

Furthermore, we found that EA played a competitive inhibitory manner against α-glucosidase with a *K*i value of 6.24 × 10^5^ mol/L, and this inhibitory process was reversible. EA also interacts with the amino acid residues including tyrosine, tryptophan, and phenylalanine of α-glucosidase and quenched the enzyme’s fluorescence *via* a static quenching mechanism. Further analysis discovered that EA could spontaneously interact with α-glucosidase at only one binding site. The results of CD spectra and AFM studies further supported the direct interactions between EA and α-glucosidase and molecular docking analysis uncovered that EA was inserted into the active site of α-glucosidase and formed intermolecular hydrogen bonds with the amino acid residues. Moreover, the α-glucosidase inhibitory activity of EA was further confirmed in Kunming mice using a sucrose-loading test. These results illuminated in detail the inhibitory mechanisms of EA against α-glucosidase both *in vitro* and *in vivo*, and further elucidated the active substance basis of anti-diabetic activity of pomegranate peel based on α-glucosidase inhibition.

## Data availability statement

The raw data supporting the conclusions of this article will be made available by the authors, without undue reservation.

## Ethics statement

The whole process conformed to the Guide for the Care and Use of Laboratory Animals, eighth edition (ISBN 10: 0-309-15396-4).

## Author contributions

RS: conceptualization, methodology, and writing—original draft. RS and LH: investigation, formal analysis, software, conceptualization, writing—review and editing, funding acquisition, project administration, and supervision. LH and HZ: methodology and software. RS and MG: investigation and formal analysis. RS and NZ: writing—review and editing. All authors contributed to the article and approved the submitted version.
