# Supplementary material for: Bioaffinity ultrafiltration coupled with HPLC-ESI-MS/MS for screening potential α-glucosidase inhibitors from pomegranate peel
Source: Front Nutr. 2022 Oct 18;9:1014862. doi: 10.3389/fnut.2022.1014862 (PMC9623087; doi:10.3389/fnut.2022.1014862)
Supplement: Supplementary file 1 [file Data_Sheet_1.docx]

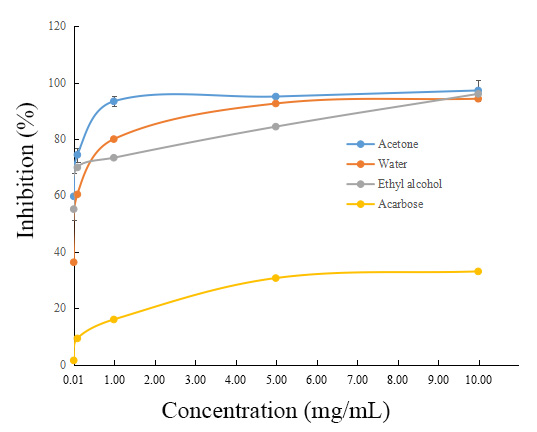


**Figure S1.** The inhibitory effect of extracts from pomegranate peel against α-glucosidase.

**
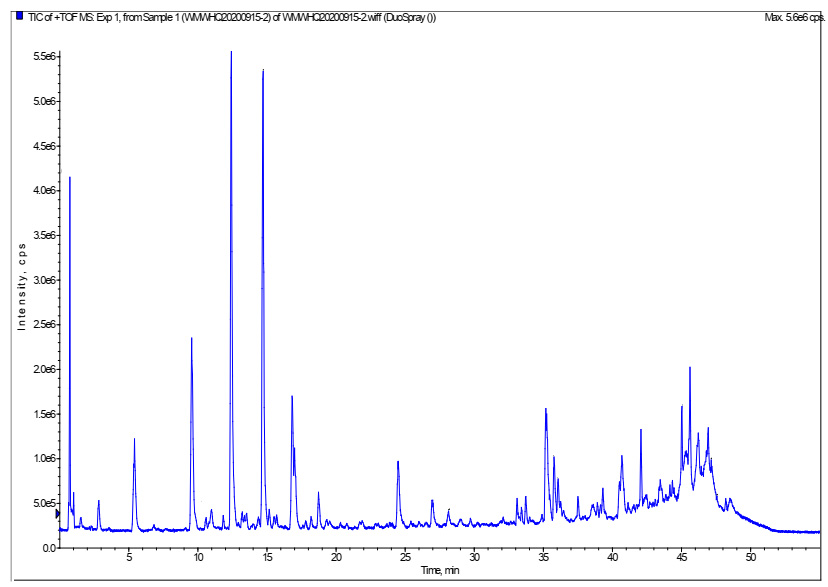
**

Ellagic acid

Nicotinic acid

Isoguanosine

Sclareol glycol

Triptolide

Sclareolide

*p*-Hydroxybenzoic acid

Kaempferol

Gallic acid

Quercetin

Resveratrol

**Figure S2.** The total ion chromatograms (TIC) of acetone extract and ion chromatograms (IC) of each active compound.


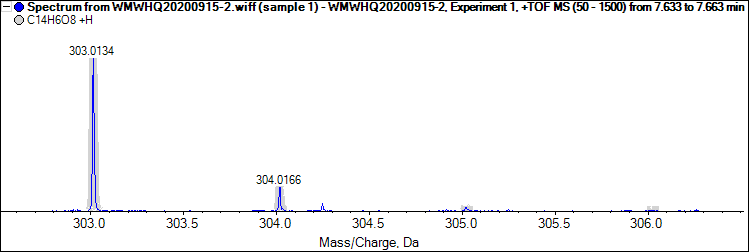


Ellagic acid


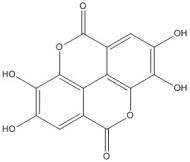


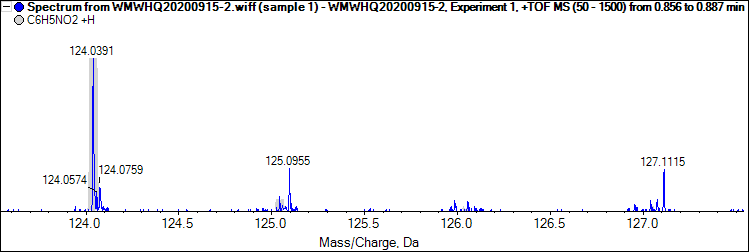


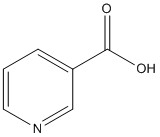


Nicotinic acid


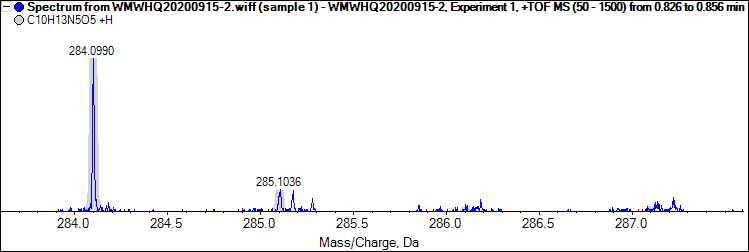


Isoguanosine


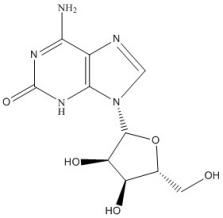


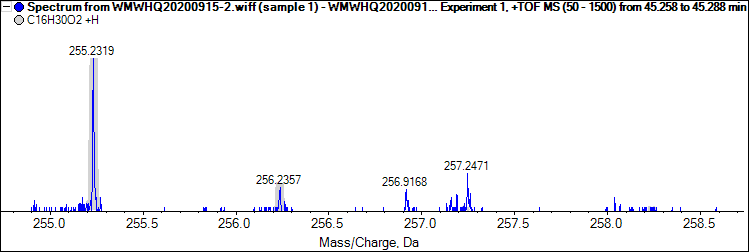


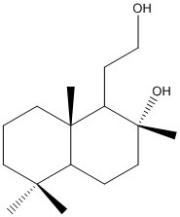


Sclareol glycol


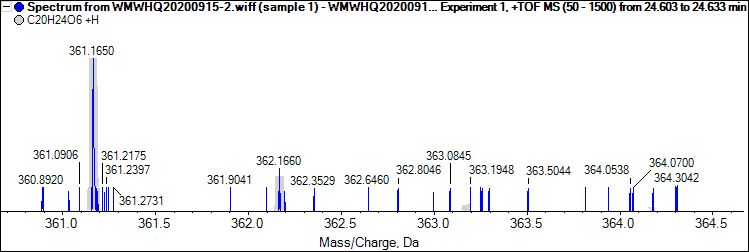


Triptolide


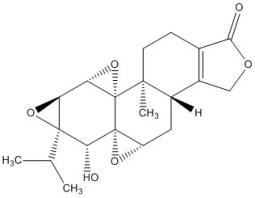


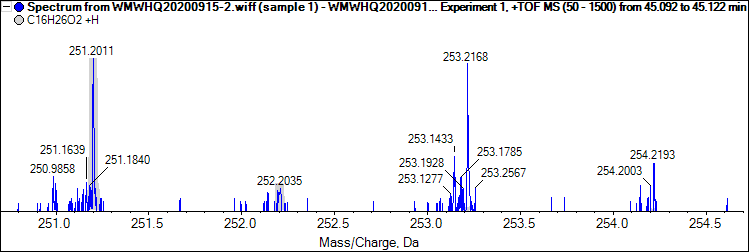


Sclareolide


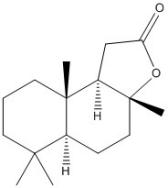


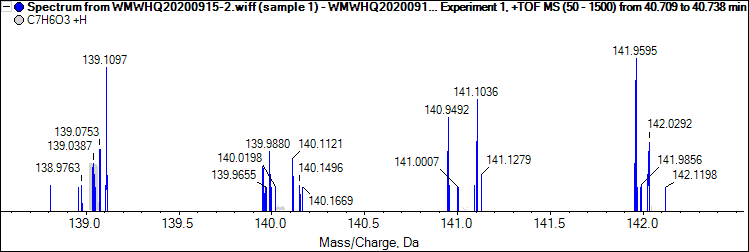


*p*-Hydroxybenzoic acid


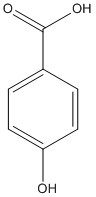


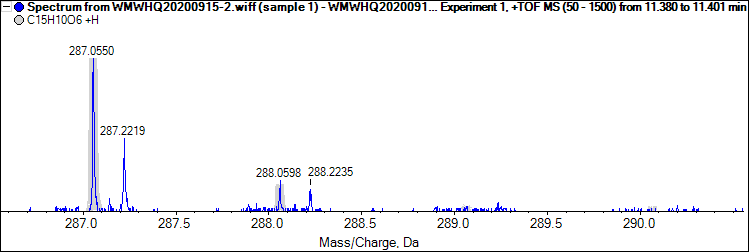


Kaempferol


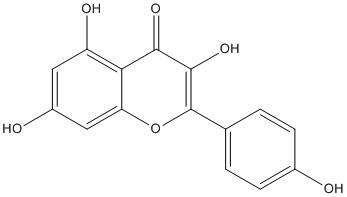


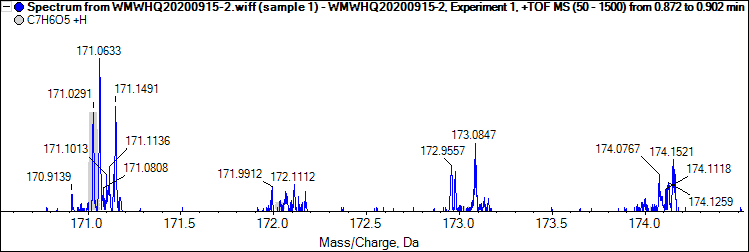


Gallic acid


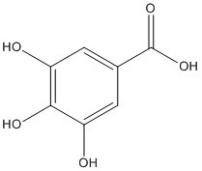


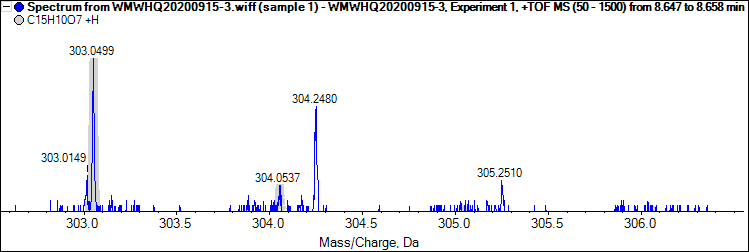


Quercetin


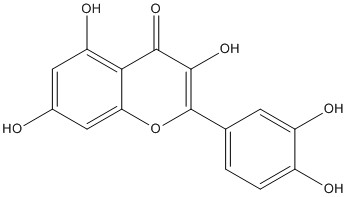


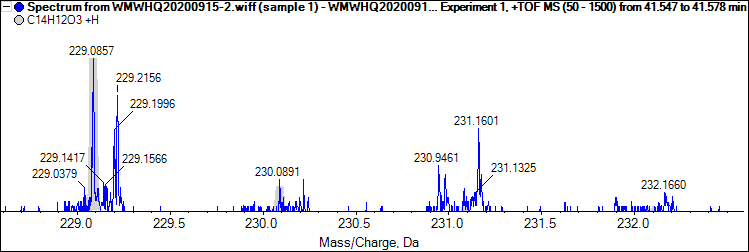


Resveratrol


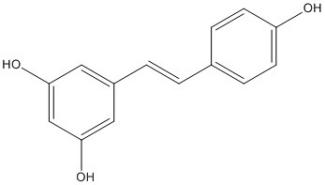


**Figure S3.** The mass spectrogram and chemical structure of each identified compound screened from the acetone extract of pomegranate peel.
